# Current trends in single‐cell RNA sequencing applications in diabetes mellitus

**DOI:** 10.1002/2211-5463.70061

**Published:** 2025-06-19

**Authors:** Seyed Sajjad Zadian, Khodakaram Jahanbin, Shekoofeh Nikooei, Marzieh Rostaminejad, Arezoo Rahimi, Pedram Abdizadeh, Behnam Alipoor

**Affiliations:** ^1^ Department of Immunology, School of Medicine Shahid Beheshti University of Medical Sciences Tehran Iran; ^2^ Department of Immunology, School of Medicine Ahvaz Jundishapur University of Medical Sciences Iran; ^3^ Student Research Committee Yasuj University of Medical Sciences Iran; ^4^ Department of Laboratory Sciences, Faculty of Paramedicine Yasuj University of Medical Sciences Iran; ^5^ Department of Internal Medicine Yasuj University of Medical Sciences Iran

**Keywords:** diabetes mellitus, diabetic nephropathy, diabetic retinopathy, single cell RNA sequencing

## Abstract

Diabetes mellitus (DM) is among the most prevalent metabolic diseases worldwide, associated with an increased risk of mortality. Although numerous studies have been conducted to uncover the cellular and molecular pathways associated with DM pathogenesis, reaching new diagnosis and treatment goals for DM requires further research. The progress in gene sequencing technologies, particularly in single‐cell RNA sequencing (scRNA‐seq), has yielded additional insights into the molecular pathways involved in the development and progression of DM. This review summarizes the latest advances and applications of RNA‐seq technologies in diabetes research, such as the characterization of single human islet and immune cells in DM, and the applications of scRNA‐seq in the treatment and early diagnosis of diabetes complications.

## Graphical abstract

Single‐cell RNA sequencing is a powerful approach to decipher the cellular and molecular landscape at a single‐cell resolution. The rapid development of this technology has led to a wide range of applications, including the detection of cellular and molecular mechanisms and the identification and introduction of novel potential diagnostic and therapeutic targets for diabetes mellitus and its related complications.

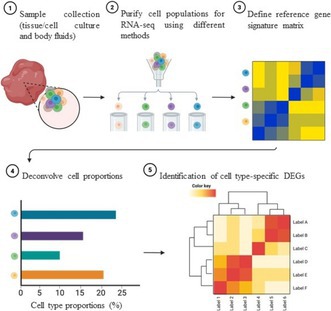

**Abbreviations**
BCRs: B cell receptors
DEGs: differentially expressed genes
DKD: diabetic kidney disease
DM: diabetes mellitus
DN: diabetic nephropathy
DR: diabetic retinopathy
eQTL: expression quantitative trait locus
EST: expressed sequence tag
FACS: fluorescence activated cell sorting
GEO: Gene Expression Omnibus
GWAS: genome wide association study
IAR T: islet antigen‐reactive CD4^+^ memory T
IFCs: integrated fluidic circuits
IRGs: immune‐related genes
RNA‐seq: RNA sequencing
scRNA‐seq: single‐cell RNA‐seq
snATAC‐seq: single‐nucleus ATAC sequencing
STRT‐seq: single‐cell tagged reverse transcription sequencing
T1DM: type 1 diabetes
T2DM: type 2 diabetes mellitus
TCRs: T‐cell receptors

Diabetes mellitus (DM), the ninth leading cause of death in the United States in 2020, is a metabolic disease characterized by abnormally high glucose levels [1]. Globally, the prevalence of diabetes in adults is estimated to be 1 in 11, of which approximately 90% is type 2 diabetes mellitus (T2DM). The prevalence of type 1 diabetes (T1DM) gradually increases from childhood, reaching a maximum at ages 4–6, and then peaking again at 10–14 [2, 3]. It has been shown that the prevalence of T2DM is approximately 9% in the overall population of the United States, but it rises to approximately 25% among individuals aged 65 years and older [4].

The pathophysiology and treatment of DM are complex and require a multitude of interventions for successful management. RNA sequencing (RNA‐seq) is a method that can be employed to address this issue. A common method currently employed to decipher transcriptional landscapes is high‐throughput RNA‐seq. This technology allows for the quantitative and comprehensive profiling of transcriptional expression on a genome scale and has a wide range of applications [5]. Single‐cell RNA‐seq (scRNA‐seq) is also known as a powerful tool for analyzing gene expression patterns and is an advanced method for investigating individual cell transcriptomes in sequenced material [6]. Currently, scRNA‐seq is being widely employed in a variety of fields, including microbiology, immunology, neurology, and oncology. The technology holds great promise in improving disease diagnosis, treatment, and prognosis [7] and has also emerged as a promising sequencing technology that allows the characterization of cellular subpopulations and the assessment of cellular heterogeneity [8]. scRNA‐seq offers an alternative way to investigate the development of DM and has the potential to introduce novel diagnostic and therapeutic possibilities within a clinical context [9, 10]. It is also beneficial to researchers in terms of observing diverse cell types across different types of DM. Furthermore, this tool can be utilized to evaluate the reproducibility and accuracy of data on various pancreatic cells and cells involved in the pathogenesis of DM. Therefore, this review summarizes the latest advances in RNA‐seq technology and recent progress in diabetes research obtained by scRNA‐seq.

## RNA sequencing

**Fig. 1:**
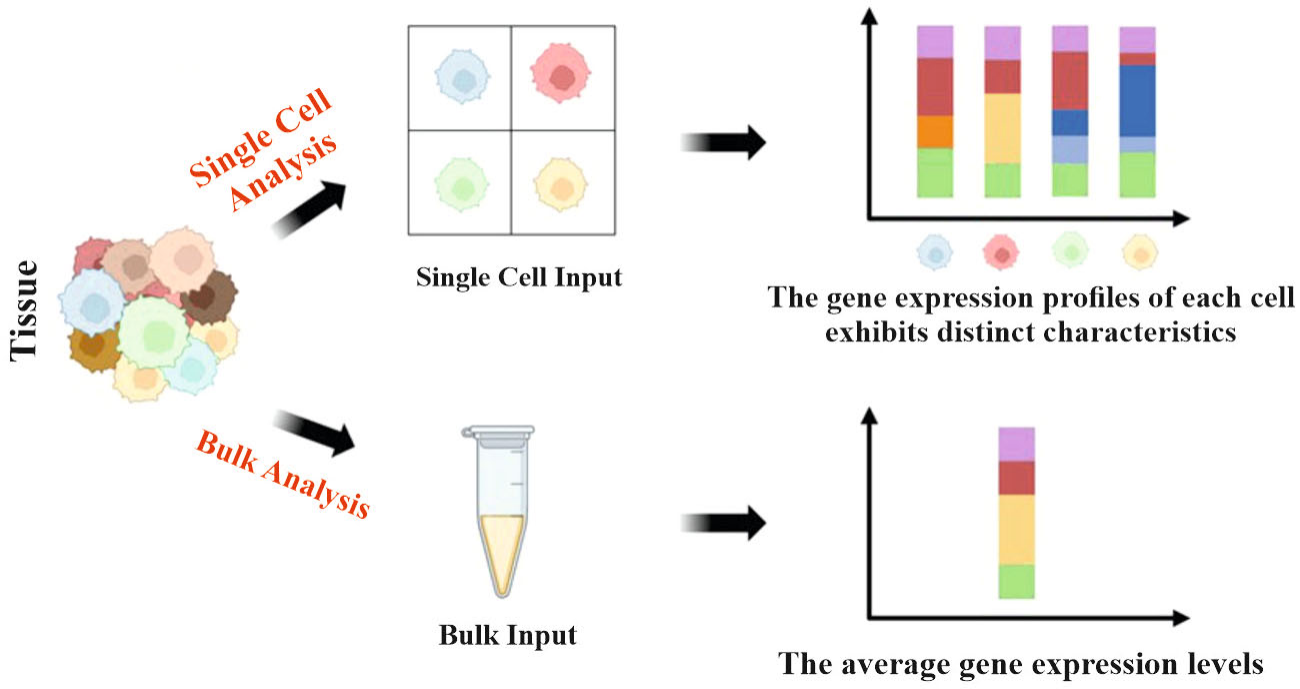
Diagram outlining the differences between bulk and single cell sequencing. scRNA‐seq facilitates a comprehensive examination of the transcriptome in individual cells from a particular sample, revealing cellular diversity that is often masked by traditional bulk RNA sequencing methods. Figure generated using BioRender.

RNA‐seq provides a more thorough insight into the genetic landscape than DNA sequencing, revealing various genetic conditions such as gene fusions, splicing variants, and mutations/indels, and providing differential gene expression patterns [11]. The most commonly used techniques in genomics have undergone a series of advances, starting with traditional bulk RNA sequencing (bulk RNA‐seq) and progressing to the widely adopted scRNA‐seq (Fig. 1) [12].

### Bulk RNA‐seq

Since the initial sequencing of the first expressed sequence tag (EST) library on a Roche 454 sequencer in 2007, bulk RNA‐seq has emerged as a valuable and extensively adopted tool for study in the field of biology [13]. For over a decade, researchers have employed this approach on RNA samples derived from cell populations to study alterations in gene expression across various tissues [14, 15]. Subsequently, systems have been optimized to accommodate various RNA types and samples with differences in initial quality. In its most basic form, bulk RNA‐seq refers to any sequencing method that works by analyzing the gene expression profile of a cell population in order to determine the presence and quantity of RNA [16]. Thus, the utilization of bulk‐based methodologies can be used to detect differences between various cell populations rather than single cells. However, bulk RNA‐seq has a variety of applications in medical research, ranging from identifying the mechanisms involved in disease pathogenesis to identifying novel potential diagnostic biomarkers and therapeutic targets [17]. Numerous RNA seq‐derived signatures have been devised and validated across a wide range of human diseases [18, 19]. Nevertheless, the translation of signature panels into healthcare settings has been restricted to just a couple of cases, mostly due to challenges in achieving reliable outcomes [20]. By looking at different RNA‐seq datasets from various studies, the prognostic potential of different biomarkers can be markedly improved [20, 21].

Certain fusion genes have shown value as diagnostic tools. For example, the fusion gene RUNX1–RUNX1T1 has been employed for the detection of acute myeloid leukemia [22]. Fusion genes such as TMPRSS2–ERG have also been identified as potential prognostic indicators in prostate cancer [23]. Therefore, numerous gene fusions have been detected using bulk RNA‐seq [24]. The therapeutic use of these findings has been limited by the high number of false positives and low detection sensitivity, but progress has been made in several studies to overcome these two challenges [25, 26]. Moreover, bulk RNA‐seq studies are limited in their ability to identify heterogeneity among individual cells and accurately obtain different gene expression profiles of specific cell subsets [11, 27]. Advances in science and technology have allowed high‐throughput single cell transcriptome sequencing technologies to steadily develop. The introduction of new technologies has increased the efficiency and sensitivity of transcriptome sequencing. These advances have facilitated unbiased, high‐throughput investigation of the transcriptome of individual cells [12].

### ScRNA‐seq

Bulk RNA‐seq has the capability to assess the average gene expression levels across cells within a given sample, detect disparities between different sample conditions, and provide an overview of highly regulated pathways, but it falls short in capturing the distinct intricacies of individual cells and the cell diversity present within tissues [12]. Moreover, some cell populations exhibit significant levels of cellular and transcriptomic heterogeneity as a result of variations in cell types or undifferentiated states [28]. The aforementioned limitations have largely been overcome with the introduction of scRNA‐seq technology, which allows comprehensive investigation of the transcriptome of individual cells in a given sample in various diseases such as colorectal cancer [29], advanced osteosarcoma [30], cervical squamous cell carcinoma [31], lung squamous cell carcinoma [32], glioblastoma [33], pancreatic tumor [34], human retinal disease [35], and osteoporosis [36]. Advances in this technology have facilitated the development of cell atlases of unparalleled detail, allowing thousands of cells to be examined simultaneously. In addition, the integration of chromatin status and multimodal analysis is now possible [37, 38].

The development of scRNA‐seq has revolutionized genome research and is a notable achievement, comparable to the success of the Human Genome Project in 2003 [39]. The Nature Methods journal appointed scRNA‐seq as a highly anticipated technology in 2013, recognizing its significance in the detection of various cell types and functions [40]. This recognition was further reinforced in 2019, when it was chosen as the technology of the year [11]. scRNA‐seq technologies typically include sample acquisition, single‐cell isolation and capture, cell lysis, reverse transcription, cDNA amplification, library preparation, high‐throughput sequencing, and data analysis [41].

The capture of single cells and the amplification of single‐cell cDNA for library construction represent the most difficult aspects of the library preparation process. Consequently, scientists have been investigating technological innovations in recent years, which have resulted in the emergence of an increasing variety of modified and improved single‐cell RNA sequencing technologies [8, 42]. Currently, various methods are employed to separate individual cells [43]. Fluorescence activated cell sorting (FACS) is the most widely utilized method; however, the isolation efficiency is relatively low [44]. Droplet‐based microfluidics, commonly referred to as ‘lab‐on‐a‐chip’, is the favored technique for single‐cell separation within a transcriptome platform. It is primarily categorized into integrated fluidic circuits (IFCs), microporous techniques, and droplet methods. This approach significantly enhances the capacity for single‐cell capture and library generation, allowing for the simultaneous analysis of thousands of cells [45, 46]. Reverse transcription and cDNA amplification are crucial processes that enhance the sensitivity and precision of scRNA‐seq. Currently, there are two prevalent amplification techniques: full‐length transcript sequencing and 3′/5′‐end transcript sequencing methods [47]. Certain protocols, including Quartz‐seq, Smart‐seq, SUPeR‐seq, and MATQ‐seq, are capable of generating full‐length transcript sequencing data, whereas others, like CEL‐seq, Drop‐seq, inDrop, 10× Genomics, and Quartz‐seq2, focus solely on sequencing the 3′‐end, and protocols such as STRT‐seq target the 5′‐end [42]. In contrast to 3′‐end or 5′‐end counting protocols, full‐length scRNA‐seq methods offer unparalleled benefits in analyzing isoform usage, detecting allelic expression, and identifying RNA editing markers, owing to their enhanced transcript coverage [48]. Systematic comparisons of the performance of several scRNA‐seq methods are well reviewed [49]. The selection of an appropriate scRNA‐seq method should be contingent upon the particular experimental context. Table 1 provides a summary of different scRNA‐seq technologies and experimental protocols.

**Table 1:** Comparison of various scRNAseq methods.

Platform/year	Isolation method	Gene coverage	Amplification method	Unique molecular identifier (UMI)	Advantages	Disadvantages	References
CEL‐seq, 2012	Micromanipulation	3′	*In vitro* transcription	–	High specificity and accuracy	Low throughput	[173]
SMART‐seq, 2012	FACS	Full length	PCR	No	Full‐length coverage	Low throughput	[174]
Quartz‐seq, 2013	FACS	Full length	PCR	Yes	High sensitivity and reproducibility	Low efficiency, high manual technical requirement	[175]
SMART‐seq2, 2013	FACS	Full length	PCR	–	High sensitivity, cell capture visualization; low noise	High manual technical requirements	[176]
MARS‐seq, 2014	FACS	3′	*In vitro* transcription	Yes	Reduced amplification bias; high reproducibility	High manual technical requirement	[177]
Cyto‐seq, 2015	Microwell platform	3′	PCR	Yes	High throughput	Costly and time‐consuming	[178]
Drop‐seq, 2015	Droplet	3′	PCR	Yes	High throughput; affordable price; fast amplification	Low sensitivity	[179]
inDrop, 2015	Droplet	3′	*In vitro* transcription	Yes	Low cost; simplified process	Inefficient cell capture rate	[180]
CEL‐seq2, 2016	FACS	3′	*In vitro* transcription	NO	High sensitivity; low cost	Low throughput	[181]
10x‐ Genomics, 2017	Droplet	3′ or 5′	PCR	Yes	Simplified process; high cell flux; high throughput	Costly and high sample requirements	[182]
MATQ‐seq, 2017	Micromanipulation	Full length	PCR	–	High sensitivity and accuracy	Low throughput	[183]
Seq‐Well, 2017	Microwell platform	3′	PCR	Yes	High throughput; user‐friendly; cost‐effective	Low cell capture efficiency	[184]
Microwell‐seq, 2018	FACS	3′	PCR	Yes	High throughput; cost‐effective; high quality	3′ bias	[185]
Quartz‐seq2, 2018	Droplet	Full length	PCR	Yes	High throughput; high sensitivity and accuracy	High manual technical requirements	[186]
SPLit‐seq, 2018	*In situ* barcoding	3′	PCR	Yes	High throughput; low cost; cell separation is not essential	Not enough genes	[187]
MARS‐seq2, 2019	FACS	3′	*In vitro* transcription	Yes	High throughput	High manual technical requirement	[188]
Microwell‐seq2, 2020	FACS	3′	PCR	Yes	High throughput; high sensitivity and stability	3′ bias	[189]
SCAN‐seq, 2020	Dilution	Full length	PCR	Yes	High sensitivity; high precision	Low throughput; expensive	[190]
Seq‐Well, 2020	Microwell platform	3′	PCR	Yes	High‐throughput; high accuracy	3′ bias	[191]
SMART‐seq3, 2020	FACS	Full length	PCR	Yes	Precise quantification; cost‐effective	High manual technical requirements	[192]
FLASH‐seq, 2022	FACS	Full length	PCR	Yes	High throughput; high sensitivity	High manual technical requirements	[193]
Smart‐seq3xpress, 2022	FACS	Full length	PCR	Yes	High throughput; high sensitivity	Time‐consuming	[194]
VASA‐seq, 2022	Plate‐based formats and droplet microfluidics	Full length	PCR	Yes	High throughput and cost‐effective	–	[195]
SCAN‐seq2, 2023	FACS	Full length	PCR	Yes	High throughput; high sensitivity	Comparatively higher in cost	[196]

## Insights from scRNA‐seq into the pathogenicity of T1DM

Multiple studies have employed scRNA‐seq to explore the complex mechanisms involved in the pathogenesis of T1DM. This progress has led to important discoveries regarding the complex area of T1DM immunology [50].

### Characterization of immune cells in T1DM through scRNA‐seq

Kallionpää *et al*. reported a significant correlation between elevated IL‐32 expressions in peripheral blood mononuclear cells (PBMCs) and both seroconversion and the development of T1DM. This association was primarily attributed to activated and highly differentiated T cells and natural killer cells [51]. Cerosaletti Linsley *et al*. uncovered a unique class of T‐cell receptors (TCRs), known as “public” chains, which transcended individual boundaries within islet antigen‐reactive CD4^+^ memory T (IAR T) cells. They showed that more than 25% of these cells shared TCR junctions, with the majority displaying specificity for islet antigen epitopes. Interestingly, they revealed that the prominence of public TCRs with shared TCRα junctions is most evident in new‐onset T1DM cases [52]. Further investigation by Eugster *et al*. into the physiological and pathogenic autoimmune processes in T1DM revealed significant differences in the TCR repertoire between healthy subjects and T1DM patients. Their findings showed how these changes are involved in the pathogenesis of the disease [53]. Performing scRNA‐seq analysis on pancreatic islets of a streptozotocin‐induced T1DM mouse model by Yang *et al*. revealed 11 distinct cell types. Their results underscored a significant reduction in mature α and β‐cells, confirmed by flow cytometry, highlighting the role of these cells in the progression of T1DM [54]. Another study showed that CD8^+^ T cells were the predominant T‐cell subset in pancreatic tissue from non‐obese diabetic (NOD) mice with T1DM. The *TRAJ23* gene showed a strong association with T1DM, and its knockout alleviated T1DM symptoms in mice. Additionally, engineered TCR‐T cells demonstrated significant cytotoxicity against β cells in T1DM [55]. Kasmani *et al*. performed scRNA‐seq and scTCR‐seq to analyze autoreactive CD8^+^ T cells in the islets and spleens of NOD mice. Their findings revealed phenotypic heterogeneity and restricted TCR gene use in CD8^+^ T cells targeting the β‐cell epitope IGRP. They identified six clusters of autoreactive CD8^+^ T cells in both the islets and spleen, including memory and exhausted cells. These insights highlight the potential for targeting specific TCR structures in therapeutic strategies for T1DM [56]. In a study performed by Elgamal *et al*., an integrated map of islet cell type‐specific gene expression was created using scRNA‐seq to explore gene activity in T1DM. This study identified significant changes in β‐cells, including the upregulation of MHC class I antigen presentation and cytokine signaling, indicating immune processes involved in T1DM [57].

Pu *et al*. used scRNA‐seq to explore immune responses in syngeneic and allogeneic islet transplantation models. They identified three macrophage clusters, including Mø‐C1, Mø‐C2, and Mø‐C3, with distinct functional roles. They also demonstrated significant upregulation of rejection‐related genes and inflammatory pathways in allogeneic transplants, with Mø‐C3 identified as a progenitor for other macrophage subsets. These findings emphasized the critical role of macrophage activation in graft rejection and offered insights into potential therapeutic targets for improving islet transplant outcomes [58]. Furthermore, Zhou *et al*. utilized scRNA‐seq to explore T‐cell dynamics in syngeneic and allogeneic islet transplantation, which revealed significant gene expression variations and immune responses across T‐cell subpopulations. They identified the activation of the interferon‐alpha pathways in memory T cells and TNF‐α signaling in regulatory and activated T cells, providing profound insight into immune interactions post‐transplantation [59].

B cells play a significant role in the pathogenesis of T1DM by presenting islet antigens to diabetogenic T cells. This antigen presentation ultimately leads to the destruction of pancreatic β‐cells [50]. It has been demonstrated that B cell receptors (BCRs) derived from naive IgD^+^ and IgM^−^ B cells can bind to insulin and cause an anergic B cell response. Conversely, BCRs derived from naive B cells display weak binding and would probably not recognize insulin under normal physiological circumstances [60]. Polyreactive antibodies have been hypothesized to significantly impact the functioning of the immune system in a beneficial manner. However, they have also been associated with the development and progression of various autoimmune disorders [60, 61, 62]. Many BCRs from healthy donors' precursor B cells have been shown to react with insulin and other self‐antigens like dsDNA, ssDNA, and nuclear proteins [63, 64]. scRNA‐seq analysis of CD19^+^IgG^+^ B cells isolated from pancreatic lymph nodes obtained from donors who tested positive for autoantibodies revealed that there were no instances of clonally expanded B cells detected in the pancreatic lymph nodes [65]. Honardoost *et al*. provided insight into the systemic immune dysregulation in T1DM through scRNA‐seq analysis of PBMCs from 46 patients and 31 matched controls. They showed 1784 dysregulated genes across 13 immune cell types, with pathways such as Wnt signaling, interferon signaling, and antigen presentation. Remarkably, many of these gene alterations were also observed in pancreatic islets, emphasizing the systemic nature of immune involvement in T1DM. Additionally, they developed a T1DM metagene z‐score to distinguish patients from controls and stratify patients into molecular subtypes. These subtypes correlated with known prognostic markers and drug responses, underscoring the utility of scRNA‐seq in developing personalized diagnostic and therapeutic strategies for T1DM [66]. Similarly, Guo *et al*. identified a novel subset of SIGLEC‐1^+^ monocytes with a strong interferon signature using scRNA‐seq on PBMCs from latent autoimmune diabetes in adults and T1DM patients. Experiments in NOD mice confirmed their role in accelerating T1DM onset. This finding demonstrates that SIGLEC‐1^+^ monocytes may serve as potential biomarkers for early diagnosis and therapeutic targets in T1DM [67]. Ji *et al*. identified 14 distinct cell types within T1DM islets. They reported that the high numbers of macrophages and T lymphocytes are correlated with severe pancreatic islet β‐cell damage. They also identified that complement C1q subcomponent subunit B could induce macrophage activation, while *NKG7* enhanced T lymphocyte activation [68]. Another study analyzed bone marrow cells in streptozotocin (STZ)‐induced T1DM mice using scRNA‐seq and revealed significant shifts in CD45^+^ immune cells, marked by an increase in bone marrow neutrophils and a decrease in B lymphocytes in T1DM mice [69]. Interestingly, scRNA‐seq also shows a larger gene expression pattern that can predict the onset of T1DM, even before autoantibodies are produced [50]. In summary, scRNA‐seq has the potential to uncover the intricate aspects of immune dysregulation in T1DM, identify specific molecular targets, and provide resources for patient classification, thereby improving both our understanding of underlying mechanisms and their clinical relevance.

### RNA‐seq of single human islet cells in T1DM

ScRNA‐seq is expected to facilitate research on the mechanisms and potential treatments of T1DM and provide valuable insights into islet cell biology. The findings of Hrovatin *et al*. in a mouse model provide critical insights into the molecular differences between various diabetes states, particularly highlighting the distinct features of autoimmune‐T1DM. Their analysis revealed molecular variations in β‐cell states, including an intermediate β‐cell phenotype between healthy controls and different diabetic models [70]. Similarly, Fu *et al*. used a mouse model of STZ‐induced diabetes to investigate the regenerative strategies of β‐cells during the development of T1DM. They found that STZ‐induced diabetes led to significant dedifferentiation of β‐cells, with some transdifferentiating into α or δ cells, a process observed in both juvenile and adult mice. Unlike the T2DM model, in which β cell proliferation occurs primarily through self‐renewal, the T1DM model shows β cell plasticity and suggests that trans‐differentiation from α‐ and δ‐cells to β‐cells may aid in islet recovery [71].

Analysis of more than 1300 individual islet cells from both non‐diabetic and T1DM donors revealed distinct gene expression patterns, particularly the absence of retinol binding protein 4 (RBP4) in surviving β cells from T1DM donors [72]. In addition, Song *et al*. used an integrated approach including expression quantitative trait locus (eQTL), genome‐wide association study (GWAS), and scRNA‐seq to investigate the pathogenic role of the CTSH gene in T1DM. They identified elevated expression of *CTSH* in the acinar cells of T1DM patients and established a strong correlation between *CTSH* and T1DM [73]. Moreover, a comparative examination of gene expression patterns in immune cells from pancreatic samples of patients revealed the upregulation of *REG1B*, *REG1A*, *INS*, *REG3A*, and *IL‐32* [51]. Zakharov *et al*. revealed transcriptional heterogeneity among immune cell populations in the pancreatic islets of non‐obese diabetic mice. They claimed that the presence of memory CD4 and cytotoxic CD8 T cells indicated the early stages of autoimmune diabetes. Their findings also showed that regulatory T cell subsets expressed genes like Foxp3 and IL10, which changed how pathogenic T cells worked [74]. The results of Qadir *et al*. showed the presence of multiple cell subpopulations, with distinct differentiation stages, including a transitional phenotype towards acinar tissue. They identified PDX1^+^/ALK3^+^/CAII^−^ progenitor‐like cells in the major pancreatic ducts (MPDs) of both type 1 and T2DM donors, regardless of disease duration. These progenitor‐like cells were found to differentiate into all pancreatic lineages, including functional β‐cells, when transplanted into immunodeficient mice [75].

ScRNA‐seq has also been used to understand how autoimmune diabetes is caused by a gain‐of‐function mutation in the *STAT3* gene. Remarkable changes were revealed within the cellular landscape of the affected mice's islets. Notably, effector CD8^+^ T cells are marked by their highly cytotoxic nature and the upregulation of genes associated with chemotaxis and cytotoxicity, including *Ccl4*, *Ccl5*, *Gzma*, *Gzmb*, and *Gzmk* [76]. Furthermore, immunostaining of the human pancreas revealed an increased presence of CADM1^+^ cells in close proximity to CD8^+^ T cells in T1DM subjects. Prior to islet infiltration, *CADM1* may affect immune cell recruitment in pancreatic acinar cells [77]. Zakharov *et al*. analyzed immune, endothelial, and mesenchymal cells extracted from pancreatic islets using sc‐RNASeq. They revealed that the primary populations of CD45^+^ cells included T cells, B cells, macrophages, conventional dendritic cells, and plasmacytoid dendritic cells [74]. Furthermore, Zakharov *et al*. discovered a subset of CD4 T cells, known as CD4‐2, which exhibited an anergic phenotype. They also identified two distinct groups of macrophages, including *Mac‐2* (*Atf3*) and *Mac‐3* (*Cxcl9*) which are activated in an inflammatory manner [74]. Collectively, scRNA‐seq has greatly enhanced our comprehension of T1DM by offering in‐depth information on gene expression, the diversity of immune cell populations, and the cellular variability present in human islets. The integration of scRNA‐seq with advanced computational tools and multi‐omics could revolutionize personalized medicine by enabling tailored therapeutic interventions based on individual cellular profiles.

## Importance and applications of scRNA‐seq in T2DM

Advances in single‐cell technology have extended research on the fundamental pathogenesis of T2DM at the gene and transcriptome levels to the single‐cell level [10]. Here, we review the advances in single‐cell technology and discuss its applications in T2DM research.

### Keratinocyte function and wound healing: skin transcriptomics

The structure of human skin is characterized by a multilayered composition that consists of various cell types, primarily keratinocytes and fibroblasts, along with a range of immune cells, melanocytes, adipocytes, and endothelial cells [78, 79]. Keratinocyte dysfunction is one of the main factors in impaired wound healing for DM patients [80]. Studies revealed that impaired wound healing is a significant complication linked to diabetes. In fact, it is estimated that between 19% and 34% of individuals with diabetes will experience a foot ulcer at some point during their condition [81]. Transcriptomic investigation of a diabetic skin‐humanized mouse model could elucidate molecular pathways involved in the impaired wound healing process [82]. scRNA‐seq has the potential to enhance our comprehension of the pathobiology associated with skin disorders and to identify critical cell populations that significantly contribute to disease progression. This methodology holds great promise for advancing drug development and improving treatment options for skin diseases [79].

The transcriptional characteristics of mast cells in patients with T2DM were explored at the single‐cell level. Liao *et al*. identified a total of 8888 cells and 8 cell clusters from skin tissue and reported that mast cells accounted for 2.7% of the total cell numbers. They also revealed that the expression levels of *ADH1C*, *PAXIP1*, *HAS1*, and *ARG1* were elevated while *PHACTR2*, *GGA1*, and *RASSF2* were reduced in mast cells from T2DM patients compared to controls [80, 83]. In a recent study, scRNA‐seq revealed the transcriptional characteristics of keratinocytes in T2DM patients at the single‐cell level. Gene set enrichment analysis revealed differential gene enrichment in signaling pathways including oxidative phosphorylation, cytokine receptor interactions, prion diseases, and other signaling pathways [84]. Furthermore, foot skin scRNA‐seq analysis identified multiple fibroblast cell clusters and elevated inflammation in the dorsal skin of DM patients and nonhealing diabetic foot ulcer specimens compared with control subjects [85]. A study by Luo *et al*. investigated mitochondrial programmed cell death in diabetic foot ulcers (DFU) through bulk RNA‐seq and scRNA‐seq. The authors identified key genes, including *BCL2* and *LIPT1*, that are downregulated in DFU, with keratinocytes as an important cell type. They also explored transcription factors such as CEBPD and IRF1, whose expression was upregulated in DFU, and validated these findings through clinical samples [86]. Ou *et al*. identified the dynamic interaction between Schwann cells (SCs) and other cell types during the early stages of wound repair. They found that SC‐derived TGF‐β3 promoted the migration of fibroblasts and keratinocytes, which enhanced wound healing [87]. In addition, Lu *et al*. demonstrated that endothelial cells from non‐healing DFUs exhibited impaired angiogenesis and downregulated expression of key genes involved in inflammation and immune signaling pathways, such as *CCND1*, *ENO1*, *HIF1α*, and *SERPINE1* [88]. It has been reported that *RAB17* expression is downregulated and angiogenesis is suppressed in human skin microvascular endothelial cells (HDMECs) derived from DFU patients. Furthermore, overexpression of *RAB17* promoted angiogenesis, increased *HIF‐1α* and *VEGF‐A* expression, and improved diabetic wound healing via the MAPK/ERK signaling pathway *in vitro* [89]. Wang *et al*. investigated the healing mechanism of DFUs using scRNA‐seq. Their analysis revealed 1948 differentially expressed genes in tissue stem cells from healing compared to non‐healing wounds, with upregulation of 1198 genes and downregulation of 685 genes. Functional enrichment analysis highlighted the role of the CCL2‐ACKR1 signaling axis in modulating endothelial cell activity and promoting wound healing in DFUs [90]. Theocharidis *et al*. used scRNA‐seq to profile 174 962 cells from DFU patients, focusing on cells from the foot, forearm, and peripheral blood. Their analysis showed that patients with healed wounds had a specific fibroblast population overexpressing *MMP1*, *MMP3*, *MMP11*, *HIF1A*, *CHI3L1*, and *TNFAIP6*, as well as increased M1 macrophage polarization [91].

Exploring the heterogeneity and functional changes of fibroblasts in DM by scRNA‐seq suggested that fibroblasts could be applied as immunomodulators in refractory diabetic wound healing, providing new ideas for the treatment of refractory diabetic wound healing [92]. Januszyk *et al*. identified a total of 384 individual cells by scRNA‐seq from human chronic wound samples. They introduced transcriptionally distinct cell clusters whose gene expression profiles corresponded to fibroblasts, keratinocytes, neutrophils, monocytes, and endothelial cells. They also revealed that the distribution of fibroblast subpopulations is altered in diabetic versus non‐diabetic cells [93]. A recent study described immune cell populations within the wound‐associated cells of STZ‐induced diabetic and wild‐type control mice using scRNA‐seq. This investigation explored the genetic differences between the various cell populations in the wound samples according to chronological order, providing a closer look into specific processes [94]. Chen *et al*. used single‐cell and RNA‐seq datasets from the Gene Expression Omnibus (GEO) database to explore macrophage‐related genes in DFU and determine potential treatment options. They identified 802 macrophage‐related genes by scRNA‐seq analysis, of which 743 were differentially expressed. Of these, *HMOX1* was revealed as a key biomarker associated with biological pathways like insulin signaling [95]. The role of M1 macrophages and immune‐related genes (IRGs) in DFUs was investigated using scRNA‐seq and high‐throughput sequencing data. Results showed that 16 different cell clusters were present in foot skin, with M1 macrophages having the highest presence in unhealed DFUs. In addition, a total of 106 M1 macrophage‐associated IRGs were identified, highlighting 25 transcription factors that regulate IRG expression [96]. scRNA‐seq could act as a powerful tool for decoding the complexity of keratinocyte function and wound healing in T2DM patients. It provides molecular insights into cellular dysfunctions, identifies therapeutic targets, and opens avenues for personalized medicine to treat chronic wounds effectively.

### RNA‐seq of single human islet cells in T2DM

Several single‐cell genomics studies revealed significant variability across islet cell types and have allowed for in‐depth studies to further understand the underlying cellular mechanisms of T2DM. Reports indicate that, unlike the T1DM model, β cells in T2DM primarily expand through self‐renewal instead of differentiating from α and δ cells [71]. It has been revealed that scRNA‐seq and ATAC‐seq profiles allow us to deconvolve alpha, beta, and delta cell populations, identify cell‐type‐specific regulatory signatures, and reveal alterations in open chromatin underlying T2DM [97, 98]. scRNA‐seq analysis suggested that the imprinted gene *Nnat* contributes to the establishment of heterogeneity in β‐cell through differential DNA methylation. This heterogeneity includes different NNAT‐positive subpopulations specialized for insulin production, which is diminished in db/db mice [99]. Suda *et al*. explored the role of Jagged1 in Notch signaling in β‐cells of obese mice and its association with β‐cell dysfunction and insulin secretion failure in T2DM. Their study indicated that Jagged1 expression followed increased Notch activity in both obese mouse models and T2DM patients. Neutralizing Jagged1 improved glucose‐stimulated insulin secretion in isolated islets. The results of this study suggest that Jagged1 is a potential therapeutic target [100]. Further research utilized scRNA‐seq to investigate β‐cell proliferation in response to fatty acids in rat islets. It has been shown that oleate enhanced β‐cell proliferation, which was linked to increased gene expression related to energy metabolism and mitochondrial activity [101]. The results of Weng *et al*. revealed marked heterogeneity in β‐cells at both transcriptomic and epigenomic levels and identified *HNF1A* as an important regulator of intra‐donor β‐cell variation. Reduced expression of *HNF1A* in T2DM β‐cells is associated with altered Na^+^ current and *FXYD2* was identified as a potential target [102]. Consistent with previous studies, Bosi *et al*. found that 210 gene expressions were upregulated and 16 gene expressions were downregulated in T2DM β‐cells. This study also highlighted key pathways involved in T2DM, including insulin deficiency, oxidative stress, and SREBP signaling [103].

*NPC1* has been identified as a key regulator of pancreatic β‐cell differentiation and function in T2DM, maintaining lysosome function and mitochondrial turnover. Loss of *Npc1* in mice has been reported to reduce β‐cell survival, β‐cell mass expansion, and insulin secretion, and to impair postnatal β‐cell differentiation [104]. Chen *et al*. identified key marker genes and transcription factors that contribute to β‐cell heterogeneity and gene expression changes in T2DM β‐cells [105]. Ma *et al*. identified two β‐cell clusters characterized by marked ferroptosis and dedifferentiation and revealed transcription factors and long non‐coding RNAs such as *MALAT1* and *MEG3* [106]. scRNA‐seq analysis of 1892 islet samples from patients with T2DM identified 11 different clusters, uncovering key pyroptosis‐related genes and pathways involved in immune infiltration and islet dysfunction [107]. Furthermore, Wang *et al*. performed snRNA‐seq and enhancer profiling in islets from lean and obese mice, which revealed heterogeneous enhancer states associated with β‐cell dysfunction in T2DM. Their analysis identified distinct gene signatures and enhancer dynamics, with metabolic stress‐induced changes linked to *H3K4me1* and *H3K27ac* modifications [108].

Patch‐seq analysis identified key genes and pathways regulating β–cell exocytosis. An analysis of function and gene expression networks identified a gene set associated with functional heterogeneity in β–cells, which can be used to predict electrophysiology [72]. It has been shown that the pathways associated with reduced β‐cell exocytosis in T2DM significantly differ from those correlated with low exocytosis in cells from non‐diabetic subjects [72]. Many sc‐RNA‐seq studies have found variable transcript enrichment across different islet cell types [9, 109, 110, 111]. Huang *et al*. highlighted four biomarkers of high diagnostic value for T2DM, including *SLC2A2*, *SERPINF1*, *RASGRP1*, and *CHL1*. Their scRNA‐seq analysis of T2DM samples revealed 13 distinct cell clusters, with fibroblasts showing significant expression of *SERPINF1*, a gene potentially regulated by *NR2F2* [112]. Yang *et al*. identified three major cell clusters, including neurons, epithelial cells, and smooth muscle cells linked to pancreatic development and insulin secretion. They also identified several key genes, including *CDKN1C* and *DLK1*, which were upregulated in T2DM samples and validated their diagnostic value [113]. A meta‐analysis of six scRNA‐seq studies from 47 metabolically healthy human islet donors and 23 donors with T2DM reported that α‐cells from T2DM donors modified genes involved in energy regulation, autophagy, cell cycle, and xenobiotic metabolism. They also suggested that the insulin secretion signaling pathway was induced in α‐cells from T2DM donors. Indeed, β‐cells from T2DM donors modified genes involved in pathways related to energy regulation, autophagy, the cell cycle, and hormone signaling pathways. Moreover, the insulin secretion signaling pathway was also induced in β‐cells from T2DM donors [114]. Motomura *et al*. discovered a diabetes‐specific transcriptome landscape of endocrine and nonendocrine cell types with subpopulations of β and α cells from the pancreatic islets of prediabetic and diabetic db/db mice, an animal model of T2DM. They showed that Anxa10‐overexpressed β cells displayed suppression of glucose‐stimulated intracellular Ca2^+^ elevation and potassium‐induced insulin secretion [115]. In summary, RNA‐seq technologies have become essential tools for characterizing human islet cells in the context of T2DM. This approach offers valuable insights into cellular diversity and transcriptional patterns, facilitating a deeper understanding of disease mechanisms and the identification of potential diagnostic and therapeutic targets.

### Transcriptomic atlas of gingival mucosa in T2DM

It has been suggested that disruption of the oral gingival barrier could induce inflammatory periodontal diseases in T2DM patients [116]. scRNA‐seq of gingiva from leptin receptor‐deficient mice (*db/db*) revealed a decreased epithelial/stromal ratio and a dysfunctional barrier. Wang *et al*. introduced inflammatory signaling between fibroblasts and neutrophils as a potential driver of diabetes‐induced periodontal damage. Collectively, they reported that the “immune‐like” fibroblasts with transcriptional diversity are involved in the innate immune homeostasis of the diabetic gingiva [117]. Moreover, scRNA‐seq analysis of gingival tissue taken from healthy and periodontitis‐affected areas of patients with and without T2DM revealed unexpected differences in gene expression among macrophages in periodontitis. It has been shown that the majority of macrophages in periodontitis express the monocyte lineage marker CD14, indicating their bone marrow lineage [118]. Agrafioti *et al*. evaluated the changes in gingival tissue affected by periodontitis using scRNA‐seq. They revealed that periodontitis reorganizes the immunological profile and function of gingival tissue and enhances inflammatory signaling in macrophages. They also observed distinct subpopulations of macrophages in periodontitis‐affected gingival tissue in T2DM patients [118]. These findings highlight the value of RNA sequencing, particularly single‐cell techniques, in analyzing the intricate cellular and molecular environment of gingival mucosa in T2DM, offering potential targets for therapeutic intervention in diabetic periodontitis.

### Sexually dimorphic transcriptome and T2DM genes

Sex differences in diabetes susceptibility, development, and progression have been reported previously, suggesting the existence of sex‐dependent diabetes‐associated genes [119, 120]. A considerable number of genes exhibiting sex bias are expressed in β cells. Li *et al*. identified 62 sex‐dependent diabetes‐altered genes through a high‐fat diet‐induced T2DM mouse model. They suggested that there is a significant difference between males and females in the molecular mechanisms mediating diabetes pathogenesis. They also showed that diabetic mice transplanted with sex‐dependent islets had impaired glucose tolerance [121]. Recent studies have revealed that female β cells have a greater ability to maintain glucose‐stimulated insulin secretion across multiple physiological and pathological conditions. In a mouse model, scRNA‐seq data demonstrated that female islets showed higher expression of genes associated with protein synthesis, folding, and processing. Moreover, islets isolated from female mice were more resistant to endoplasmic reticulum stress induction by thapsigargin. Indeed, female islets were more capable of maintaining glucose‐stimulated insulin production and secretion during endoplasmic reticulum stress compared to males [122]. Qadir *et al*. investigated sex differences in human pancreatic islets from 52 T2DM and non‐diabetic donors using scRNA‐seq and snATAC‐seq. Results showed that in non‐diabetic donors, sex differences were primarily related to sex chromosomes, whereas in T2DM donors, sex differences spanned both sex chromosomes and autosomal genes. Mitochondrial respiration was suppressed in female β cells, whereas insulin secretion was impaired in male β cells, suggesting an association between female mitochondrial dysfunction and T2DM [123]. A study by Yong *et al*. used both perifusion and scRNA‐seq data to examine how the genetic program, secretory function, and regulatory mechanisms of male and female pancreatic endocrine cells differ. Their findings revealed that female endocrine cells exhibit higher secretion capacity and molecular signatures distinct from T2DM compared to males [124]. These investigations emphasize the utility of scRNA‐seq in uncovering cellular heterogeneity and sex‐specific molecular pathways in T2DM, thereby facilitating the development of targeted treatments and personalized medical strategies.

### Immune cell dysregulation in T2DM

Immune cells, including CD14 monocytes, cytotoxic CD4 T cells, effector memory CD8 T cells, gamma delta (γδ) T cells, and B cells, have been reported to exhibit pro‐inflammatory features in T2DM patients. Intermediate monocytes showed high expression of *MHC* class II genes, and T cells showed enhanced cytotoxic activity and increased clonal expansion [125]. Zhang *et al*. investigated the role of immune cell‐mediated chronic inflammation in T2DM and identified inflammatory markers in circulating immune cells as key predictors of insulin resistance. Their scRNA‐seq also revealed that *MAFB* expression was increased mainly in non‐classical monocytes, suggesting its role in mediating chronic inflammation in T2DM [126].

## Application of scRNA‐seq in DM complications

### Diabetic nephropathy (DN)

Despite recent therapeutic advances, diabetic nephropathy (DN) remains one of the most important causes of death in diabetic patients. Advanced scRNA‐seq could serve as a powerful tool to provide information on complex cell‐to‐cell communication and explore novel biomarkers in diabetic kidney disease (DKD) [127].

#### Cell clusters in scRNA profiles of mice and human kidney tissue

Fu *et al*. identified five distinct cell populations, including glomerular endothelial, mesangial, podocytes, immune, and tubular cells, by scRNA‐seq analysis of isolated glomerular cells from streptozotocin‐induced diabetic mice. They reported that the number of glomerular endothelial cells and immune cells increased while podocytes and mesangial cells decreased in diabetic kidneys compared to controls. Macrophages were the predominant cells in the immune cluster of diabetic glomeruli [128]. Another scRNA‐seq analysis of kidney immune cells in the T1DM mouse model by Fu *et al*. revealed transcriptional heterogeneity in early DKD. Their study identified dynamic changes in macrophage subsets, with increased expression of inflammatory and anti‐inflammatory genes over time. They found that macrophages exhibit a continuum of activation and differentiation, shifting towards an undifferentiated, M1‐like inflammatory phenotype as the disease progresses [129]. scRNA‐seq was also used to characterize the glomerulus from healthy and diseased mice, including models of nephrotoxic serum nephritis, diabetes, doxorubicin toxicity, and CD2AP deficiency. As a result, various types of glomerular cells and novel marker genes, including those for mesangial cells, endothelial cells, and podocytes, were identified. The study also revealed cell‐type‐specific responses to injury, particularly highlighting the activation of the Hippo pathway in podocytes after nephrotoxic injury [130]. To study T2DM at the single‐cell level, Xie *et al*. developed a conditional cell‐specific network (CCSN) from single‐cell transcriptional data of human islet samples. Their analysis identified *ATP6AP2* as a key gene involved in insulin regulation and DN. Their results also highlighted the identification of *NFATC2* as a potential biomarker for DN [131].

scRNA‐seq of renal cells from T2DM mice revealed that mesangial and proximal tubule (PT) cells have the highest gene expression changes in the early stages of DN. Indeed, renal cells from T2DM mice show a decreased proportion of proximal tubular cells and an enrichment of glomerular cell types. Proximal tubule and mesangial cells are the most sensitive cells to hyperglycemia and could induce glucose‐dependent and glucose‐independent downstream pathways in the kidneys through their response to stress [132]. In line with this finding, a reduced percentage of proximal tubular cells has been shown in db/db mice compared to db/m mice [133]. Wu *et al*. identified an average of 1167 unique genes and 2105 transcripts per cell in kidney samples from a DKD mouse model. They also revealed that the proximal tubule cells have the highest number of differentially expressed genes during the progression of DKD [134]. Moreover, integrative omics analyses of ATAC‐seq and RNA‐seq data revealed the epigenetic memory underlying the deregulation of key target genes in T2DM‐PT that may contribute to sustained renal dysfunction in DKD [135].

Maxwell *et al*. used scRNA‐seq of mouse models to determine the regulatory role of Set7 lysine methyltransferase in DKD. They showed that *Set7* knockout improves glomerular structure and albuminuria by regulating glomerular endothelial cell populations through transcriptional modulation of insulin growth factor binding protein 5 [136]. In a recent study, scRNA‐seq analysis identified macrophages as the most increased cell type in DKD and introduced four immune marker genes, including *SYK*, *ITGB2*, *FCER1G*, and *VAV1* as potential diagnostic and therapeutic targets for DKD [137]. Similarly, Zhang *et al*. performed a comprehensive scRNA‐seq and transcriptomic analysis of kidney cells in DKD, revealing eight distinct cell types, with macrophages showing the highest intercellular interactions. They identified *SLIT3*, *PDE1A*, and *CFH* as key immune‐associated genes closely related to DKD. Immune infiltration analysis also indicated significant associations between these genes and macrophages, T cells gamma delta, and resting mast cells [138]. Guo *et al*. investigated the role of macrophages (Mφs) in DN using snRNA‐seq. They identified changes in the Mφ populations, particularly a depletion of protective Mφs expressing *CD163*, *MRC1*, *PTH2R*, *PDE4D*, and *CUBN* in DN. This depletion was associated with altered macrophage functions, including their interaction with podocytes, contributing to podocyte dysfunction and apoptosis [139]. Menon *et al*. identified 31 distinct cell clusters, including glomerular endothelial cells from 22 268 single‐cell profiles of renal biopsies by analyzing scRNA‐seq data. Their study focused on the phenotype of endothelial cells in the renal vascular bed and introduced molecular subtypes of glomerular disease. Indeed, two focal segmental glomerulosclerosis (*FSGS*) patient subgroups were identified based on alpha‐2 macroglobulin (*A2M*) expression, which was associated with proteinuria remission rates and long‐term renal outcomes [140]. Similarly, Latt *et al*. used scRNA‐seq to analyze urine samples from FSGS patients. They identified immune cells, predominantly monocytes, and renal epithelial cells in urine, with monocytes showing M1 and M2 phenotypes and shedding podocytes expressing epithelial‐to‐mesenchymal transition (EMT) marker genes. Comparison of transcriptome data from renal biopsies revealed that immune and EMT gene signatures in urine were more highly expressed in *FSGS* than in minimal change disease (MCD) [141].

scRNA‐seq has been used to investigate hyperglycemia‐induced transcriptional changes across multiple tissues, including the kidney, in a streptozotocin‐induced type 1 diabetes mouse model. The results identified conserved fibroblast alterations, such as an increase in myeloid‐like fibroblasts and elevated levels of decorin, a fibrosis regulator linked to DN progression [142]. Zhou *et al*. investigated endothelial cell (EC) injury in DKD using scRNA‐seq to analyze 5464 ECs from diabetic and non‐diabetic mice at three time points. They identified 13 different EC phenotypes, highlighting functional zonation along nephrons and differential regulation of pathways like EIF2 signaling and oxidative phosphorylation. Their study also revealed that dynamic changes in alternative splicing are associated with DNA repair [143]. Furthermore, single‐cell and transcriptomic analysis of thick ascending limb (TAL) cells in DN introduced seven potential biomarkers, including *COBL*, *PPARGC1A*, and *THSD7A* [144]. A recent investigation identified four distinct clusters of proximal tubules, including PTS1/2, PTS3, PT containing AQP4 expression, and proliferative PT. There were fewer PT and PTAQP4^+^ cells dividing in the kidneys of db/db mice, which caused the renin‐angiotensin system to stop working properly in early DKD [127]. It has been shown that mitochondrial dysfunction could reduce quantity, impair oxidative function, and disrupt fuel transition in DKD. Interestingly, it has been reported that the mitochondrial contents of db/db mice showed distinct trends in different parts of the nephron [145]. Analysis of unbiased snRNA‐seq identified 11 kidney cell types and 4 immune cell types in cryopreserved human diabetic kidney samples from three control and three early DN samples. This data revealed that the proximal convoluted tubules are enriched in genes involved in the regulation of IL‐8 production, albumin transport, and modulation of angiogenesis [146].

#### Application of scRNA‐seq in the treatment and diagnosis of DN

scRNA‐seq analysis revealed that the gene expression profile of kidney cells in T2DM db/db mice changed following treatment with angiotensin receptor blockers (irbesartan) and sodium‐glucose co‐transporter 2 inhibitors (dapagliflozin). Data revealed that irbesartan exerts its therapeutic effect through anti‐inflammatory and anti‐fibrotic mechanisms, while dapagliflozin influences mitochondrial function, fatty acid metabolism, and ATP consumption in proximal tubular cells [133]. Integrative analysis of ATAC‐seq and RNA‐seq data of kidney samples from DKD‐ mice revealed that the transcriptional alterations of DN and the intervention with dapagliflozin were significantly influenced by chromatin remodeling. Shen *et al*. identified novel targets for dapagliflozin within critical signaling pathways, including glucolipid metabolism, oxidative stress, and xenobiotic and endobiotic metabolism. They proposed several candidate genes, such as *UDP* glucuronosyltransferase 1 family, *Dock2*, and *Tbc1d10c*, along with transcriptional regulators like *KLF6* and *GFI1* that may be linked to DN and the restorative effects of dapagliflozin [147].

Wu *et al*. revealed a heterogeneous response of all renal cell types to both monotherapy and combination therapies. They showed that sodium‐glucose co‐transporter 2 inhibitors could induce fasting mimicry and hypoxia responses by targeting the S1 segment of the proximal tubule [134]. Lu *et al*. used scRNA‐seq to analyze the kidneys of rats treated with empagliflozin, an SGLT2 inhibitor. The results demonstrated that empagliflozin treatment significantly reduced renal interstitial fibrosis, glomerulosclerosis, and inflammation. They reported that empagliflozin inhibits the polarization of pro‐fibrotic CD206^+^CD68^+^ M2 macrophages by targeting mitophagy and mTOR pathways and attenuating inflammatory signals from CD8^+^ effector T cells [148].

scRNA‐seq has been suggested to be a powerful tool that can provide information on complex cell‐to‐cell communication and introduce novel biological markers in human diseases such as diabetes and its related complications [127, 149]. Tsai *et al*. revealed that the urinary levels of ceruloplasmin are higher in db/db mice and are positively correlated with proximal tubule injury markers, including kidney injury molecule‐1 (KIM‐1)/creatinine and neutrophil gelatinase‐associated lipocalin. They also reported that the expression of *SEMA3C* is higher in mesangial cells of db/db mice than in db/m mice, and high glucose treatment increased *SEMA3C* expression in mesangial cells. They also demonstrated that the urinary secreted phosphoprotein 1 (SPP1)/creatinine level is positively correlated with proximal tubule injury markers, and T2DM patients had a higher urinary SPP1/creatinine level than normal individuals. These consistent results were also found in humans [127]. Finally, findings obtained from scRNA‐seq of human kidney samples from 23 DKD patients and 10 healthy controls demonstrated that proximal tubules, connecting tubules, and principal cells as likely cellular sources of increased tissue expression of matrix metalloprotease 7 (*MMP7*) showed the strongest association with both fibrosis and eGFR [150].

### Diabetic retinopathy (DR)

Due to the complicated structure of the retina, the pathogenesis of DR is not yet fully understood. However, RNA seq‐based assays have tried to explain the complex network of molecular and cellular changes that underlie DR and provide clues to potential diagnostic and therapeutic targets for DR [151, 152]. Sun *et al*. revealed that four stress‐inducible genes, including *Cirbp*, *Rmb3*, *Mt1*, and *Mt2*, are commonly induced in most types of retinal cells in streptozotocin‐induced diabetic mice. They also demonstrated that diabetes upregulates the expression of inflammatory factor genes in retinal microglia and stimulates the expression of immediate early genes in retinal astrocytes [152]. Another study revealed a novel transcriptional landscape and heterogeneity of retinal cells in T2DM mice. In this regard, it has been reported that DR could influence cell‐type‐specific genes and alter intercellular communication [153]. scRNA‐seq of retinal tissue from 12‐week‐old wild‐type and Akimba mice revealed alternations in cell metabolism and ribosomal gene expression, activation of immune system pathways, and redox and metal ion homeostasis [154]. Li *et al*. demonstrated an increased proportion of retinal pigment epithelial cells (RPEs) in STZ mice using scRNA‐seq analysis [155]. Zhang *et al*. constructed a single‐cell atlas of DR to analyze differences between DR and healthy retinal samples. Their findings showed reduced numbers of bipolar cells, Müller glia, retinal pigment epithelial cells, and cone photoreceptors, as well as an increase in pericytes, rod photoreceptors, anaplastic cells, and microglia from DR tissues. They also revealed that oxidative stress and inflammation promote disease progression and contribute to visual impairment [156]. Chen *et al*. identified heterogeneity of bipolar cells (BCs) and a novel RBC subtype (Car8_RBC) in the mouse retina. Certain BCs and rod cells showed significant upregulation of AC149090.1 in T2DM mice. Their data also revealed that BCs are particularly vulnerable to diabetes [157]. Data obtained from a recent study demonstrated that adipocyte enhancer‐binding protein 1 signaling could modulate pericyte‐myofibroblast transformation, and its downregulation could inhibit scar tissue formation in advanced DR [158]. Moreover, based on previous publications, scRNA‐seq analysis revealed that retinaldehyde‐binding protein 1 is a promising therapeutic target for DR. Overexpression of this protein in Müller glia has been implicated in DR‐associated neurovascular degeneration [153].

Yao *et al*. identified a diabetes‐specific retinal EC population characterized by high inflammatory gene expression and significant involvement of sphingolipid metabolism, particularly alkaline ceramidase 2 (*ACER2*). Their findings showed elevated ceramide levels in different stages of DR, indicating that overexpression of ACER2 may prevent endothelial barrier disruption and vascular leakage [159]. Similarly, Ren *et al*. investigated EC heterogeneity in DR using scRNA‐seq. Their study identified three distinct EC subpopulations in the retina and highlighted the role of the mitochondria‐localized glutamic acid‐rich protein (*Mgarp*) gene in DR pathogenesis [160]. Chen *et al*. revealed decreased expression of AQP1 in diabetic retinal samples and high‐glucose‐treated ECs, which corresponded to decreased vascular diameter and perfusion in a hyperglycemic zebrafish model [161]. Another study found that *p53* expression was significantly increased in retinal endothelial cells and its inhibition reduced senescence markers such as SA‐β‐Gal in human retinal microvascular endothelial cells. Furthermore, *p53* was shown to promote the ubiquitination and degradation of FoxO3a through the upregulation of the ubiquitin‐conjugating enzyme UBE2L6 [162]. Haliyur *et al*. identified four different cell states in the vitreous, including T cells, B cells, myeloid cells, and neutrophils, with T cells comprising 91.6% of vitreous cells compared to 46.2% in peripheral blood. Their analysis also indicated the activation of CD4^+^ and CD8^+^ memory T cells and unique ligand‐receptor interactions in the vitreous [163]. Wang *et al*. employed an integrative approach combining scRNA‐seq, non‐negative matrix factorization (NMF), machine learning, and AlphaFold 2 to explore the molecular mechanisms of PDR. Their scRNA‐seq identified five major cell types and 14 subtypes in PDR, highlighting significant gene expression differences. *ALKBH1*, *PSIP1*, and *ATP13A2* were identified as key players in PDR pathogenesis, with predictive models achieving high accuracy [164]. Single‐cell analysis was applied to investigate the role of Müller cells in the DR rat model and human patients. The analysis identified distinct Müller cell clusters with unique gene expression profiles. In particular, Rho gene expression was elevated in certain clusters, suggesting a phagocytic response to damaged photoreceptor cells in the DR microenvironment. Co‐expression of *Rho* and *PDE6G* in these clusters highlighted their role in maintaining retinal integrity during DR [165]. Moreover, Liao *et al*. examined the role of circulating immune cells in diabetic DR. Their study showed that PBMCs from type 1 DR patients disrupted the normal function of retinal endothelial cells. scRNA‐seq analysis identified unique gene expression patterns in PBMCs from DR patients, highlighting the upregulation of *JUND* [166]. Interestingly, Dong *et al*. investigated the mechanisms by which high glucose levels influence retinal vascular endothelial cells through RNA transcriptome sequencing. Their study revealed that bone morphogenetic protein 4 could act as a potential therapeutic target for DR [167]. Finally, He *et al*. demonstrated the therapeutic potential of GLP‐1 receptor agonists (GLP‐1 RAs) in diabetic DR through a retrospective cohort study of 1626 T2DM patients. scRNA‐seq and immunostaining revealed that *GLP‐1R* expression in retinal endothelial cells was downregulated under diabetic conditions. Treatment with GLP‐1 RAs restored receptor expression, improved vascular integrity, and reduced retinal degeneration in diabetic mice [168].

### scRNA‐seq in diabetes‐associated atherosclerosis

Atherosclerosis continues to be a primary cause of death among diabetic patients, even with improvements in cardiovascular treatment [169]. scRNA‐seq has provided remarkable insights into the plasticity and transcriptional reprogramming of endothelial cells (EC) as the disease advances [170]. Zhao *et al*. analyzed EC‐enriched cells from the mouse heart and aorta after 12 weeks of feeding a standard chow or a diabetogenic high‐fat diet with cholesterol. They identified eight unique clusters of endothelial cells, three of which exhibited markers indicative of endothelial‐to‐mesenchymal transition (EndMT). This transformation towards a mesenchymal state was associated with decreased fatty acid oxidation and increased regulation of the extracellular matrix, implying that metabolic reprogramming contributes to atherosclerotic alterations. The diabetic state heightened the heterogeneity of endothelial cells, with transcriptomic analyses revealing elevated inflammatory pathways and apoptosis signaling in comparison to control groups [171]. A study conducted in 2024 involving streptozotocin‐induced diabetic Apoe^−/−^ mice demonstrated an increase in the activity of the activator protein‐1 (AP‐1) complex in aortic endothelial cells subjected to turbulent flow conditions [172]. This mechanosensitive transcription factor facilitated endothelial activation and the formation of foam cells. Notably, pharmacological inhibition of AP‐1 led to a 42% reduction in atherosclerotic lesions in diabetic mice, achieving this through its dual impact on endothelial dysfunction and the uptake of lipids by macrophages [172].

Through the identification of key cell types, gene markers, and signaling pathways, scRNA‐seq significantly improves our comprehension of DM pathogenesis and opens new avenues for targeted therapies and early diagnostic biomarkers for diabetes complications such as DN, DR, and atherosclerosis. The combination of scRNA‐seq with multi‐omics strategies presents substantial opportunities for creating targeted treatments aimed at reducing vision impairment in individuals with diabetes. Nevertheless, obstacles such as high expenses, data intricacy, and technical constraints continue to hinder its broader implementation.

## Conclusion

**Fig. 2:**
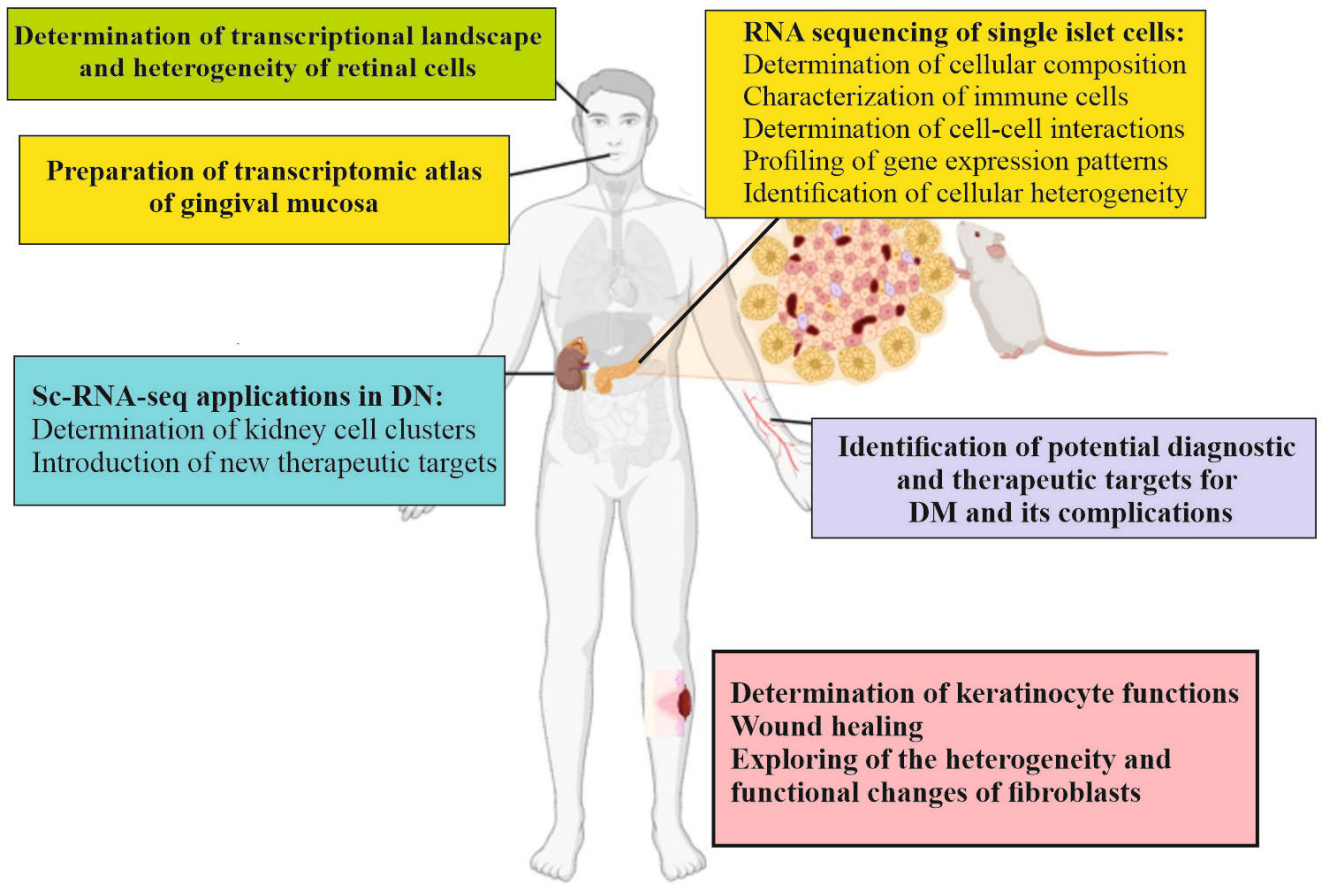
Schematic illustration of scRNA‐seq applications in DM. scRNA‐seq applications in DM include the following: (a) determination of cellular composition and cell–cell interactions of islet cells and identification of their cellular heterogeneity; (b) preparation of a transcriptomic atlas of islet cells, keratinocytes, retinal cells, and kidney cells; and (c) identification and introduction of new potential diagnostic and therapeutic targets for DM and its related complications. Figure generated using CorelDRAW and BioRender.

scRNA‐seq is a powerful approach to decipher the cellular and molecular landscape at a single‐cell resolution. The rapid development of this technology has led to a wide range of applications, including the detection of cellular and molecular mechanisms of human diseases and the introduction of new potential diagnostic and therapeutic targets. By reviewing the relevant literature, we summarize the latest advances in RNA‐seq technology in the study of DM pathogenesis and its related complications (Fig. 2). Collectively, the applications of scRNA‐seq in DM include: (a) determination of cellular composition and cell–cell interactions of islet cells and identification of their cellular heterogeneity; (b) preparation of transcriptome atlases of islet cells, keratinocytes, retinal, and kidney cells; and (c) identification and introduction of novel potential diagnostic and therapeutic targets for DM and its related complications.

## Conflict of interest

The authors declare no conflict of interest.

## Author contributions

SSZ and BA designed the project and wrote the paper, KJ and SN wrote the paper, SN and MR acquired the data, AR and PA revised the manuscript. All authors reviewed and approved the final article version.
